# Exploratory Bivariate Genome-Wide Analysis in Northern Chinese Twins Suggests Potential Loci at 2q33.1 Harboring *SPATS2L* for Lung Function and Fasting Plasma Glucose

**DOI:** 10.3390/genes17030251

**Published:** 2026-02-24

**Authors:** Xinyu Zhang, Tong Wang, Chunsheng Xu, Weijing Wang, Xiaocao Tian, Dongfeng Zhang

**Affiliations:** 1 Department of Epidemiology and Health Statistics, The College of Public Health, Qingdao University, NO. 308 Ning Xia Street, Qingdao 266071, China; 2 Qingdao Municipal Center for Disease Control and Prevention, Qingdao 266033, China

**Keywords:** lung function, fasting plasma glucose, bivariate GWAS

## Abstract

Background: Chronic obstructive pulmonary disease (COPD) and type 2 diabetes mellitus (T2DM) frequently coexist, yet the shared genetic variants underlying these conditions remain poorly understood. This study aimed to investigate shared genetic variants underlying lung function and glucose levels in middle-aged Chinese twins. Methods: In this exploratory analysis, we reanalyzed genotype data from a previously published northern Chinese twin sample, including 139 dizygotic and 238 monozygotic twin pairs from the Qingdao Twin Registry. Lung function traits (FEV1, FVC, and FEV1/FVC) and fasting plasma glucose (FPG) were jointly analyzed using a twin-based bivariate genome-wide association approach, followed by functional annotation and gene-based analyses. Variants showing suggestive associations were further examined in an independent UK Biobank Chinese sample. Results: A significant negative correlation between the FEV1/FVC ratio and FPG was revealed. The analysis identified 29 SNPs reaching genome-wide significance, with association signals primarily clustering at the 2q33.1 locus. Functional annotation indicated that most associated variants were non-coding, with several SNPs overlapping regulatory elements annotated to the *SPATS2L* locus. Gene-based analysis further supported the involvement of *SPATS2L* in the shared genetic architecture of the two traits. In the validation analysis, seven variants at the 2q33.1 locus showed nominal associations with consistent effect directions. Conclusions: This exploratory bivariate analysis provides evidence supporting shared genetic variants underlying pulmonary function and glucose regulation and offers insight into the genetic basis of COPD–T2DM comorbidity.

## 1. Introduction

Chronic obstructive lung disease (COPD) and type 2 diabetes mellitus (T2DM) are the most common chronic conditions worldwide, exerting a considerable influence on substantial impairments in quality of life and increased mortality [1,2]. Lung function measures, including forced expiratory volume in one second (FEV1), forced vital capacity (FVC), and the FEV1/FVC ratio, together with fasting plasma glucose (FPG), form the clinical basis for the diagnosis and assessment of COPD and T2DM, respectively. Accumulating epidemiological evidence supports a bidirectional association between the two conditions. Individuals with COPD are at elevated risk of developing glucose metabolic disorders [3,4], whereas individuals with T2DM are more likely to experience impaired lung function [5,6]. This comorbidity may be partly attributable to shared environmental risk factors, such as smoking and obesity, as well as common pathogenic mechanisms, including systemic inflammation, oxidative stress, and metabolic dysregulation [7,8,9], providing biological plausibility for comorbidity between COPD and T2DM.

In addition to environmental factors, genetic factors have been shown to contribute substantially to the development of both COPD and T2DM. Previous studies have shown that lung function exhibits moderate to high heritability, with reported estimates of approximately 0.3–0.9 [10,11], whereas the heritability of fasting plasma glucose has been estimated to range from 0.24 to 0.68 [12,13]. Moreover, a twin study conducted in Denmark reported a genetic correlation of 43% between COPD and T2DM [14], and Zhu et al. reported significant genetic correlations between FEV1, FVC, and T2DM [15]. Our research team has previously conducted a univariate GWAS identifying variants associated with fasting plasma glucose, including signals at the *SPATS2L* locus [16]. In parallel, previous univariate GWAS have identified hundreds of genetic loci associated with lung function [16] and glucose level [17], separately. The direct evidence for the shared genetic variants jointly influencing both traits remains limited yet. Bivariate genome-wide association studies (GWASs) jointly model multiple phenotypes to identify potential pleiotropic genetic variants, rather than relying on the overlap of results from independent univariate analyses. Compared with univariate GWASs, multivariate GWASs improve the ability to detect pleiotropic effects and genetic variants with small effect sizes [18,19]. Moreover, integrating GWAS with a twin-based design could effectively control for population stratification and gene–environment(rGE) correlation, thereby enabling a clear distinction between direct and indirect genetic effects [20].

Therefore, building upon our previous univariate GWAS, we designed a bivariate twin study using data from Qingdao, China, to: (1) estimate the genetic correlation between lung function and glucose and (2) identify shared genetic variants, genes, and pathways underlying these traits.

## 2. Materials and Methods

### 2.1. Samples

The main sample and genotype data analyzed in the present study were derived from the same Qingdao Twin Registry [21] as our previously published univariate GWAS [13]. Details of information on sample collection have been described in the previous literature [22]. We obtained questionnaires, physical examination, and blood test data from all twin pairs. Exclusion criteria included pregnancy or lactation, current use of insulin or other hypoglycemic medications, and incomplete measurements of lung function, fasting plasma glucose, or key covariates, or lacked complete co-twin pair information. The final sample consisted of 377 complete twin pairs, including 238 monozygotic (MZ) twins and 139 dizygotic (DZ) twins. All participants provided written informed consent. The study was approved by the Regional Ethics Committee of the Qingdao Centers for Disease Control and Prevention Institutional Review Boards (Decision reference number: 2012–01) and was conducted in accordance with the Declaration of Helsinki.

### 2.2. Phenotype

Lung function was evaluated using an electronic handheld spirometer (Micro 0102, Micro Medical Ltd., Rochester, UK), which measured FEV1 and FVC in liters. The FEV1/FVC ratio was expressed as FEV1 divided by FVC. After at least 8 h of fasting, fasting plasma glucose (FPG) levels were measured using a semiautomatic analyzer (Hitachi 7600, Hitachi High-Technologies Corporation, Tokyo, Japan). Details of the measurement procedure were described elsewhere [11]. To improve distributional normality, FEV1, FVC, the FEV1/FVC ratio, and FPG were rank-based normalized using Blom’s method.

### 2.3. Genotyping, Quality Control, and Imputation

Genotyping was performed on genomic DNA obtained from DZ twins using the Infinium Omni2.5Exome-8 v1.2 BeadChip (Illumina, San Diego, CA, USA). Variants located on autosomes and the X chromosome were included in the analysis. In the initial quality control procedures [23,24], after quality control, SNPs with a locus missing rate < 5%, call rate > 98%, minor allele frequency (MAF) > 0.05, and no deviation from Hardy–Weinberg equilibrium (HWE, *p* > 1 × 10^−5^) were retained for analysis. Moreover, untyped SNPs were imputed using IMPUTE2 (v2.3.2) [25] with the 1000 Genomes Project Phase 3 East Asian panel as the reference. After post-imputation quality control (INFO > 0.9, MAF > 0.05, and HWE *p* > 1 × 10^−5^), 5,628,083 SNPs were retained for the final analysis.

### 2.4. Statistical Analysis

#### 2.4.1. Heritability

We applied a bivariate Cholesky decomposition model [26] using the Mx software to estimate the genetic correlations between lung function indices and FPG, adjusting for age, sex, body mass index (BMI), and smoking status (yes/no). Variance components were estimated using the classical ACE twin model, in which phenotypic variation was attributed to additive genetic (A), shared environmental (C), and unique/non-shared environmental (E) factors. Model fit was evaluated using likelihood ratio tests in conjunction with Akaike’s Information Criterion (AIC) [27]. Likelihood ratio tests were used to compare nested models, and AIC was applied to guide model selection when differences were not statistically significant (*p* ≥ 0.05) [28].

#### 2.4.2. SNP-Based Analysis

We utilized GEMMA [29] to examine the associations between lung function-FPG pairs and SNP genotypes. The analysis was adjusted for age, sex, BMI, smoking status (yes/no), the first five principal components, and the relatedness and population structure. Genome-wide significance was defined at *p* < 5 × 10^−8^, with *p* < 1 × 10^−5^ indicating suggestive associations. The GCTA-COJO software was employed to identify independent SNPs from significant association signals using a 5 Mb window and an LD collinearity threshold of r^2^ = 0.9 [30]. Regional association patterns derived from the genome-wide association study (GWAS) results were visualized using LocusZoom. Ensembl Variant Effect Predictor (VEP) [31] was used to annotate the putative molecular consequences of all suggestive SNPs. In addition, all suggestive SNPs were queried in the UK Biobank Plasma Proteomics Project [32] (UKB-PPP; https://metabolomics.helmholtz-munich.de/ukbbpgwas/ (accessed on 7 February 2026)) to identify protein quantitative trait loci (pQTLs). SNPs located in non-coding regions were further interrogated using EnhancerDB to determine their associated enhancer elements. Expression quantitative trait locus (eQTL) mapping was conducted using cis-eQTL information from GTEx v7. Only significant SNP–gene pairs with a false discovery rate (FDR) ≤ 0.05 were retained.

#### 2.4.3. Gene-Based Analysis

Gene-based association analysis was performed using MAGMA (v1.08) as implemented in FUMA [33]. SNPs were assigned to protein-coding genes from GENCODE v19 annotations (hg19) based on gene boundaries extended by ±50 kb. For each gene, SNP-level association statistics were aggregated into a gene-based test while accounting for linkage disequilibrium (LD) between SNPs using the 1000 Genomes Project Phase 3 East Asian reference panel. A total of 19,980 protein-coding genes constituted the background gene set, among which genes with at least one mapped SNP were included in the gene-based tests. Gene-based statistical significance was assessed using a Bonferroni-adjusted threshold of *p* < 2.50 × 10^−6^ (0.05/19,980), while *p* < 0.05 was considered nominal.

### 2.5. Validation Analysis

**Figure 1:**
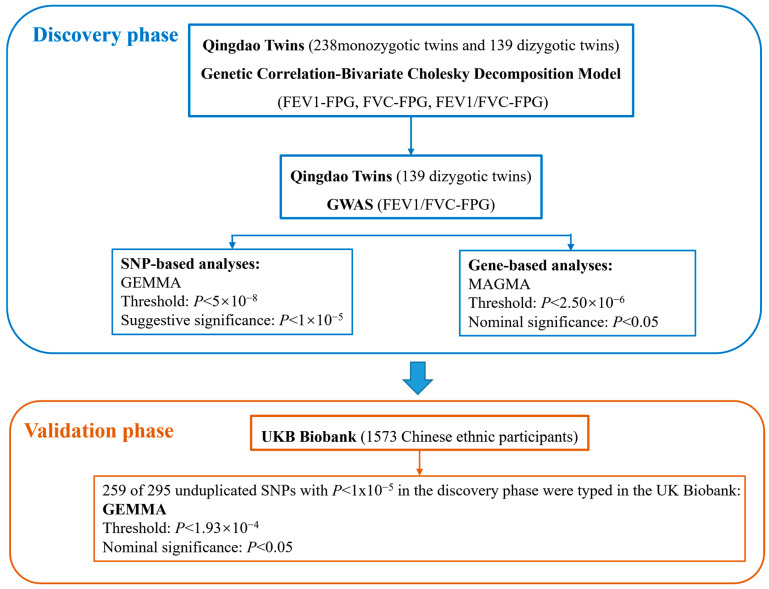
Overview of the study design and analytical workflow.

Associations identified in the discovery stage were examined in an independent dataset using phenotype and genotype data from the third UK Biobank release (Application ID: 95715). FEV1/FVC was calculated by dividing FEV1 by FVC from spirometry blow. Blood glucose levels in the UK Biobank were measured from blood samples using standard biochemical assays. Procedures for data collection, genotyping, and imputation have been reported previously [34,35]. We restricted the sample to the Chinese ethnic background (*n* = 1573). The same GEMMA software was applied to conduct validated analysis with adjustment for BMI, smoking status, and the first ten principal components. A total of 259 SNPs with *p* < 1 × 10^−5^ in the discovery analysis were typed in the UK Biobank and advanced to validation analysis. A Bonferroni-adjusted threshold of *p* < 1.93 × 10^−4^ (0.05/259) was applied, with *p* < 0.05 indicating nominal significance. Figure 1 summarizes the overall study design and analysis workflow. Statistical analyses were conducted in R (version 4.2.0).

## 3. Results

### 3.1. Basic Characteristics

Table S1 summarizes the baseline characteristics of 377 twin pairs (median age, 50 years), including 238 monozygotic and 139 dizygotic pairs. Among all participants, 363 were males, and 391 were females. Median (interquartile range) values in total sample for FEV1, FVC, FEV1/FVC ratio and FPG were 2.01 (0.87), 2.18 (0.90), 0.96 (0.12), and 5.10 (1.21), respectively. Among DZ twins, the corresponding median (interquartile range) values were 2.05 (0.97), 2.20 (0.90), 0.97 (0.10), and 5.13 (1.30), respectively.

### 3.2. Genetic Correlations

The genetic correlations between lung function and FPG are shown in Table 1. The best fitting Cholesky decomposition model (AE) indicated a significant negative genetic correlation between FEV1/FVC ratio and FPG, estimated at −0.206 (95%CI: −0.392, −0.027). However, the genetic correlation between FEV1 or FVC and FPG was not statistically significant. Therefore, we only conducted a bivariate GWAS on measures of FEV1/FVC and FPG.

### 3.3. SNPs-Based Genome-Wide Association Study

**Figure 2:**
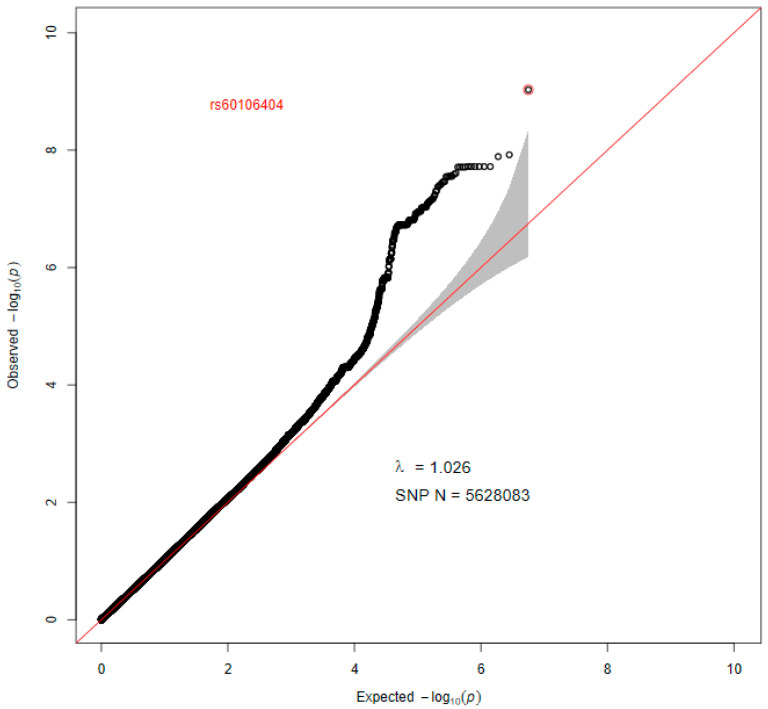
Quantile–quantile (Q–Q) plot illustrating the results of the bivariate genome-wide association analysis for the FEV1/FVC ratio–FPG. The expected −log10(*p*) values under the null hypothesis, derived from the chi-square distribution, are shown on the x-axis, and the observed −log10(*p*) values are shown on the y-axis. Each point represents a single SNP, with the leading variant highlighted. The diagonal reference line indicates the distribution expected in the absence of association. The shaded area represents the 95% confidence interval under the null hypothesis.

**Figure 3:**
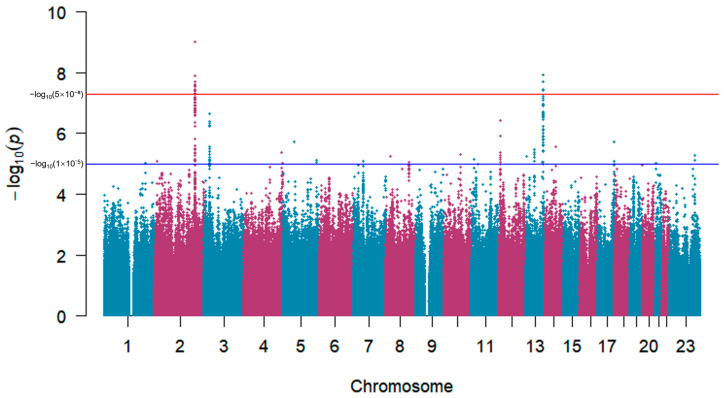
Manhattan plot of the bivariate genome-wide association study for the FEV1/FVC ratio–FPG. The x-axis represents autosomal chromosomes and the X chromosome, and the y-axis shows −log10(*p*) values for statistical significance. Each dot represents an SNP. A total of 29 SNPs exceeded the genome-wide significance threshold.

As the Q-Q plot (Figure 2) shows, there was no evidence of population stratification effects (λ = 1.026) in our results. The Manhattan plot (Figure 3) illustrates that 29 SNPs exceeded the genome-wide significance threshold, while 295 SNPs showed suggestive associations (*p* < 1 × 10^−5^, Table S2).

Table 2 presents the 29 SNPs reaching genome-wide significance. Of these variants, 19 SNPs were located on chromosome 2q33.1, as illustrated in Figure S1. Including the most strongly associated SNP, rs60106404 (*p* = 9.34 × 10^−10^), the majority of SNPs at the 2q33.1 locus were annotated as non-coding variants, including intronic non-coding transcript variants, upstream gene variants, and intergenic variants. Among these variants, eight SNPs overlapped the enhancer regions enh34140 (chr2:201,115,349–201,126,575) and enh112925 (chr2:201,102,066–201,109,495), which have been annotated as regulatory elements linked to the *SPATS2L* gene. Moreover, eQTL mapping identified six SNPs associated with the expression of *FTCDNL1* and *C2orf69* across skeletal muscle, subcutaneous adipose tissue, lung tissue, and the brain hippocampus. Detailed eQTL mapping results are provided in Table S3. In addition, two SNPs (rs3036485 and rs4673944) were intron variants located within the *SPATS2L* gene. Furthermore, 10 SNPs were located on chromosome 13q33.2, as shown in Figure S2. All of them were non-coding variants, including intronic non-coding transcript variants and downstream gene variants. Nine of them are located within an lncRNA transcript (ENSG00000295604). The conditional analysis identified three independent SNPs, including rs60106404 (*p*-adjusted = 5.29 × 10^−9^) and rs4516415 (*p*-adjusted = 1.20 × 10^−7^) in 2q33.1 locus and rs9558417 (*p*-adjusted = 8.46 × 10^−8^) in 13q33.2 locus. At present, no corresponding pQTL information was identified for the suggestive SNPs.

Table S3 summarizes the eQTL mapping results. One SNP, four SNPs, and seven SNPs were associated with the expression of the *FTCDNL1* gene in skeletal muscle, subcutaneous adipose tissue, and lung tissue, respectively. In addition, 1 SNP and 29 SNPs were associated with *SPATS2L* expression in whole blood and lung tissue, respectively. Furthermore, 45 SNPs and 34 SNPs were associated with the expression of *C2orf69* in skeletal muscle and whole blood, respectively.

### 3.4. Gene-Based Analysis Results

Gene-based association analysis using MAGMA identified *SPATS2L* (*p* = 4.53 × 10^−8^) as significantly associated with FEV1/FVC ratio and FPG. A total of 1059 genes exceeded the nominal significance threshold of *p* < 0.05, as presented in Table S4. Table 3 displays the top 20 genes ranked by *p*-value, including the *C2orf69* gene identified in eQTL analysis. These genes are mainly involved in inflammation, metabolism, and neuro-regulatory processes.

### 3.5. Validation Results

Of the SNPs showing suggestive association (*p* < 1 × 10^−5^) in the discovery phase, 259 variants were available in the UK Biobank and therefore evaluated in the validation analysis. Although none of these SNPs reached the Bonferroni-adjusted significance criterion (*p* < 1.93 × 10^−4^), seven SNPs reached nominal significance (*p* < 0.05). These SNPs showed consistent directions between the discovery and validation stages, and all were located on chromosome 2q33.1. As shown in Table S5, three SNPs (rs296801, rs295118, and rs10497859) overlapped the enhancer region enh59005, which has been annotated as a regulatory element linked to the *SPATS2L* gene.

## 4. Discussion

In this study, we applied a bivariate genetic model to estimate the genetic correlation between lung function and FPG. We identified a significant negative genetic correlation between FEV1/FVC ratio and FPG (*r*G = −0.206), suggesting a shared genetic basis underlying the two traits. Given that FEV1/FVC ratio and FPG are key indicators for the diagnosis of COPD and T2DM, respectively, this finding supports the presence of common genetic mechanisms linking the two diseases. Consistent with our results, a previous study reported a genetic correlation of approximately 43% between COPD and T2DM [14]. Therefore, further investigation of shared genetic variants influencing both COPD and T2DM is warranted.

In this exploratory bivariate GWAS, we examined shared genetic variants associated with the FEV1/FVC ratio and FPG in northern Chinese twins. We identified 29 SNPs located at the 2q33.1 and 13q33.2 loci that reached genome-wide significance. Conditional analysis indicated that only three of these variants represented independent association signals, suggesting that the majority of the identified SNPs reflect correlated signals within a limited number of genomic loci. Functional annotation based on Ensembl VEP showed that the most genome-wide significant variants were non-coding, including intronic non-coding transcript variants and intergenic or upstream variants. Among the 19 SNPs at the 2q33.1 locus, two were located within the *SPATS2L* gene region, while several others overlapped enhancer regions annotated as regulatory elements linked to *SPATS2L*, indicating that this locus affects gene expression rather than direct alterations of protein structure or function. Consistent with these observations, our previous univariate GWAS identified SNPs within the *SPATS2L* gene associated with fasting plasma glucose [13], and prior GWASs have reported associations between *SPATS2L* and lung function [16,36,37,38]. *SPATS2L* has been reported to be involved in cellular responses to oxidative stress [39] and may encode the precursor of thymulin [40], a peptide with anti-inflammatory properties [41]. Oxidative stress and inflammation are recognized pathological features of both COPD and T2DM, providing a biological context in which variation at this locus may be relevant to the observed associations. SNPs at the 13q33.2 locus were predominantly located within an lncRNA transcript (ENSG00000295604), whose potential functional relevance requires further investigation.

eQTL analysis revealed that 12 SNPs linked to *FTCDNL1* expression across skeletal muscle, subcutaneous adipose tissue, and lung tissue, tissues relevant to both FEV1/FVC ratio and FPG. Previous studies have also linked *FTCDNL1* to lung diseases [42] and T2DM [43,44]. In addition, 30 SNPs were associated with *SPATS2L* expression in whole blood and lung tissue, providing further regulatory annotation for this locus. Moreover, associations between 79 SNPs and *C2orf69* expression were detected in skeletal muscle and whole blood. *C2orf69* is a key regulator of mitochondrial function in humans [45]. No significant pQTLs were identified for the associated SNPs, indicating that the current evidence for these loci is primarily based on transcriptomic-level regulatory annotation rather than direct effects on protein abundance. The absence of detectable pQTLs in current plasma proteomic datasets may reflect limitations in protein coverage or tissue specificity, and protein-level validation will require future studies. In addition, 266 SNPs showed suggestive association. Of these, 121 SNPs mapped to six genes: *SPATS2L*, *NRG3* [46,47,48,49,50], *SGCG* [51,52,53], *KCNIP1*, *DOCK9* [13,36,38,54], and *TRPS1* [55,56], all of which have been implicated in previous studies of lung function or COPD and FPG or T2DM.

In the UK Biobank validated analysis, seven SNPs showed nominal associations with FEV1/FVC ratio and FPG. All of these SNPs were located on chromosome 2q33.1, a region that also harbored several genome-wide significant variants in the discovery analysis. Among them, three SNPs overlapped enhancer regions annotated as regulatory elements linked to the SPATS2L gene, as described above.

In the gene-based analysis, *SPATS2L* showed a statistically significant association, further supporting its involvement in both FEV1/FVC ratio and FPG. In addition, several genes reached nominal significance and have been previously implicated in lung function and glycemic traits. *HCAR1*, a lactate receptor, is a well-established regulator of inflammation and lung injury [57] and has also been shown to attenuate inflammasome-mediated pancreatic injury [58], potentially influencing glucose homeostasis. *TNFRSF1A* encodes inflammatory mediators associated with COPD [59,60], and receptor-mediated apoptosis driven by *TNFRSF1A* may contribute to the progression of β-cell dysfunction from prediabetes to T2DM [61]. *KNTC1* has been associated with lung function [54] and HbA1c levels [62] in previous GWASs. *JUND* has been linked to both lung function [63] and T2DM [64], possibly due to its protective role against p53-dependent senescence and apoptosis. *C2orf47* has been reported to be associated with FEV1/FVC ratio [38] and FPG [13]. *HCAR2* mediates potent anti-inflammatory effects across multiple tissues [65]. has been associated with lung function [54] in previous GWAS and inhibits insulin secretion; notably, it is downregulated in pancreatic β-cells in T2DM [66,67]. *RAD18* has been linked to lung function [16], and its polymorphisms may increase susceptibility to T2DM [68]. Finally, *RAB3A* plays a critical role in stimulating insulin secretion in pancreatic β-cells [69,70] and is involved in respiratory system–related biological pathways.

The present study has several advantages. First, to our knowledge, this study represents an initial effort to jointly investigate genetic variants associated with lung function and glucose metabolism in Chinese adults using a bivariate genome-wide approach, thereby providing an exploratory perspective on the shared genetic architecture underlying COPD and T2DM. Second, we employed a twin-based bivariate GWAS design, which enables the identification of direct genetic effects while effectively controlling for population stratification and passive rGE owing to the shared genetic background and early-life environment of twins. Nevertheless, several limitations should be acknowledged. First, the discovery analysis was conducted in a relatively modest twin cohort. Although the twin design offers advantages in controlling for shared genetic background and environmental factors, it does not fully meet the sample size requirements typically needed to detect modest genetic effects underlying complex traits in genome-wide association analyses. Accordingly, the present findings are interpreted as exploratory and hypothesis-generating rather than definitive and warrant cautious interpretation. Further validation in larger, independent, and population-representative cohorts will be required to establish their robustness and generalizability. Second, the validation analysis provided limited confirmatory support for the discovery findings. Given the evaluation of multiple variants, some nominal associations may arise by chance, and the current validation results do not yet provide sufficiently consistent evidence for firm confirmation. Therefore, the validation stage is interpreted as preliminary, and additional validation in larger and well-characterized cohorts will be essential to assess the reproducibility of the identified loci. Nevertheless, the consistency of association signals within a limited number of loci supports the relevance of these regions for further investigation. Third, the present findings are based on association analyses and do not allow causal inference. Accordingly, the results should be interpreted with caution, and additional studies will be needed to further clarify potential causal relationships. Fourth, as our study sample was drawn from a relatively limited number of Han Chinese twins from the Qingdao region, the generalizability of our findings may be restricted. Future studies in larger and more diverse populations are warranted to confirm the robustness and broader applicability of these results.

## 5. Conclusions

In conclusion, this exploratory bivariate GWAS identified multiple genetic variants, genes, and pathways jointly associated with the FEV1/FVC ratio and FPG. These findings offer a preliminary overview of shared genetic signals between pulmonary and metabolic traits and highlight candidate loci for future validation, contributing to ongoing efforts to understand the genetic basis of COPD–T2DM comorbidity.

## Figures and Tables

**Table 1:** Genetic correlations between lung function and fasting plasma glucose.

Phenotypic	Model	rG (95%CI)	rC (95%CI)	rE (95%CI)	AIC	χ2	Δdf	*p*
Full models								
FEV1-FPG	ACE	−0.071 (−0.237, 0.219)	−1.000 (−1.000, 1.000)	0.068 (−0.061, 0.194)	867.414			
FVC-FPG	ACE	0.085 (−0.177, 0.458)	−1.000 (−1.000, 1.000)	−0.002 (−0.129, 0.125)	851.320			
FEV1/FVC-FPG	ACE	−0.378 (−1.000, 1.000)	1.000 (−1.000, 1.000)	0.105 (−0.020, 0.227)	1019.801			
Best-fit models								
FEV1-FPG	AE	−0.095 (−0.234, 0.045)	−−	0.070 (−0.058, 0.195)	861.469	0.055	3	0.997
FVC-FPG	AE	−0.075 (−0.209, 0.060)	−−	0.0105 (−0.114, 0.135)	847.432	2.112	3	0.550
FEV1/FVC-FPG	AE	−0.206 (−0.392, −0.027)	−−	0.102 (−0.020, 0.220)	1014.979	1.179	3	0.758

FEV1: forced expiratory volume in 1 second; FVC: forced vital capacity; FPG: fasting plasma glucose; rG: genetic correlation coefficient; rC: common environmental correlation coefficient; rE: special environmental correlation coefficient; AIC: Akaike’s Information Criterion; df: free degree; 95%CI: 95% confidence interval.

**Table 2:** The twenty-nine SNPs (*p* < 5 × 10^−8^) significantly associated with the FEV1/FVC ratio–FPG in the genome-wide association study.

SNP	Band	Chr:BP	*p*-Value	Consequence	Located Gene	Functional Annotation Gene
rs60106404	q33.1	2:201118971	9.34 × 10^−10^	Intron variant. Non-coding transcript variant.	*ENSG00000297701*	*SPATS2L* *
rs9558417	q33.2	13:105545977	1.20 × 10^−8^	Downstream gene variant.		
rs4516415	q33.1	2:201129608	1.28 × 10^−8^	Intergenic variant	*--*	*FTCDNL1* ^#^/*C2orf69* ^#^
rs1409465	q33.2	13:105554903	1.90 × 10^−8^	Intron variant. Non-coding transcript variant.	*ENSG00000295604*	
rs4567577	q33.2	13:105556018	1.90 × 10^−8^	Intron variant. Non-coding transcript variant.	*ENSG00000295604*	
rs4614581	q33.2	13:105556214	1.90 × 10^−8^	Intron variant. Non-coding transcript variant.	*ENSG00000295604*	
rs9558420	q33.2	13:105555501	1.90 × 10^−8^	Intron variant. Non-coding transcript variant.	*ENSG00000295604*	
rs4772656	q33.2	13:105557207	1.90 × 10^−8^	Intron variant. Non-coding transcript variant.	*ENSG00000295604*	
rs9586670	q33.2	13:105556817	1.90 × 10^−8^	Intron variant. Non-coding transcript variant.	*ENSG00000295604*	
rs12469091	q33.1	2:201124735	1.94 × 10^−8^	Upstream gene variant.		*SPATS2L* */*FTCDNL1* ^#^/*C2orf69* ^#^
rs4233994	q33.1	2:201129211	1.94 × 10^−8^	Intergenic variant.	*--*	*FTCDNL1* ^#^/*C2orf69* ^#^
rs12474914	q33.1	2:201130210	1.94 × 10^−8^	Intergenic variant.	*--*	*FTCDNL1* ^#^/*C2orf69* ^#^
rs13007517	q33.1	2:201129729	1.94 × 10^−8^	Intergenic variant.	*--*	*FTCDNL1* ^#^/*C2orf69* ^#^
rs3036485	q33.1	2:201194504	2.48 × 10^−8^	Intron variant.	*SPATS2L*	
rs13022984	q33.1	2:201116067	2.57 × 10^−8^	Intron variant. Non-coding transcript variant.	*ENSG00000297701*	*SPATS2L* */*FTCDNL1* ^#^/*C2orf69* ^#^
rs4673814	q33.1	2:201108133	2.79 × 10^−8^	Intron variant. Non-coding transcript variant.	*ENSG00000297701*	*SPATS2L* *
rs1369842	q33.1	2:201108987	2.79 × 10^−8^	Intron variant. Non-coding transcript variant.	*ENSG00000297701*	*SPATS2L* *
rs295119	q33.1	2:201117944	2.79 × 10^−8^	Intron variant. Non-coding transcript variant.	*ENSG00000297701*	*SPATS2L* *
rs295134	q33.1	2:201110223	2.83 × 10^−8^	Intron variant. Non-coding transcript variant.	*ENSG00000297701*	
rs10931893	q33.1	2:201114652	2.83 × 10^−8^	Intron variant. Non-coding transcript variant.	*ENSG00000297701*	*FTCDNL1* ^#^
rs34467224	q33.1	2:201133705	3.43 × 10^−8^	Intergenic variant.		
rs12865613	q33.2	13:105557462	3.44 × 10^−8^	Intron variant. Non-coding transcript variant.	*ENSG00000295604*	
rs9558423	q33.2	13:105558266	3.57 × 10^−8^	Intron variant. Non-coding transcript variant.	*ENSG00000295604*	
rs1590590	q33.2	13:105559940	3.80 × 10^−8^	Intron variant. Non-coding transcript variant.	*ENSG00000295604*	
rs10804097	q33.1	2:201104924	3.90 × 10^−8^	Intron variant. Non-coding transcript variant.	*ENSG00000297701*	*SPATS2L* *
rs10931892	q33.1	2:201104997	4.10 × 10^−8^	Intron variant. Non-coding transcript variant.	*ENSG00000297701*	*SPATS2L* *
rs842830	q33.1	2:201131124	4.20 × 10^−8^	Intergenic variant.		
rs4673944	q33.1	2:201198471	4.89 × 10^−8^	Intron variant.	*SPATS2L*	
rs4233996	q33.1	2:201131228	4.98 × 10^−8^	Intergenic variant		

* indicates enhancer elements; # indicates expression quantitative trait loci (eQTLs); genes annotated with Ensembl gene IDs represent RNA genes (long non-coding RNAs, lncRNAs).

**Table 3:** The top 20 genes from gene-based analysis by using MAGMA.

GENE	CHR	START	STOP	NSNPS	*p*
***SPATS2L***	2	201120604	201396986	451	4.53 × 10^−8^
***HCAR1***	12	123054824	123265390	298	5.70 × 10^−6^
***PDE4C***	19	18268771	18416229	339	1.32 × 10^−5^
***TNFRSF1A***	12	6387923	6501280	216	3.41 × 10^−5^
***PLEKHG6***	12	6369602	6487672	227	3.64 × 10^−5^
***KIAA1683***	19	18317908	18435319	276	5.03 × 10^−5^
***SGSH***	17	78130515	78244722	263	9.37 × 10^−5^
***KNTC1***	12	122961793	123160943	230	1.26 × 10^−4^
***C2orf76***	2	120009801	120174404	352	1.43 × 10^−4^
***JUND***	19	18340563	18442432	248	1.49 × 10^−4^
***LSM4***	19	18367040	18484084	242	1.87 × 10^−4^
***AC096582.1***	7	45852684	45956045	186	2.58 × 10^−4^
***C2orf47***	2	200770040	200923263	246	2.65 × 10^−4^
***TYW5***	2	200744698	200870459	170	3.31 × 10^−4^
***C2orf69***	2	200725979	200870658	189	3.48 × 10^−4^
***SPO11***	20	55854815	55969050	46	3.57 × 10^−4^
***HCAR2***	12	123135840	123237890	150	4.12 × 10^−4^
***RAD18***	3	8767088	9055457	763	4.74 × 10^−4^
***MTRNR2L3***	20	55883496	55984878	53	4.92 × 10^−4^
***RAB3A***	19	18257594	18364884	206	5.81 × 10^−4^

## Data Availability

The dataset analyzed during the current study is available in the European Nucleotide Archive (ENA) repository (Accession No. PRJEB23749, website https://www.ebi.ac.uk/ena/browser/view/PRJEB23749 (accessed on 7 February 2026)). The genome-wide association summary statistics generated in this study are publicly available via Zenodo (https://doi.org/10.5281/zenodo.18549036).

## Supplementary Materials

The following supporting information can be downloaded at: https://www.mdpi.com/article/10.3390/genes17030251/s1. Table S1: Basic characteristics of the twin sample by sex. Table S2: All SNPs with suggestive significance associated with the FEV1/FVC ratio–FPG in the genome-wide association study. Figure S1: Regional association plot showing association signals around the 2q33.1 locus for the genome-wide association study of the FEV1/FVC ratio–FPG. Figure S2: Regional association plot showing association signals around the 13q33.2 locus for the genome-wide association study of the FEV1/FVC ratio–FPG. Table S3: Significant SNP–gene pairs associated with the FEV1/FVC ratio–FPG identified by eQTL analysis. Table S4: Nominally significant genes associated with the FEV1/FVC ratio–FPG identified by gene-based analysis. Table S5: Nominal significance SNPs (*p* < 0.05) of FEV1/FVC ratio-FPG in UK Biobank validation analysis.

## Abbreviations

The following abbreviations are used in this manuscript:

COPD	Chronic obstructive pulmonary disease
T2DM	Type 2 diabetes mellitus
FEV1	Forced expiratory volume in one second
FVC	Forced vital capacity
FPG	Fasting plasma glucose
GWAS	Genome-wide association study
MZ	Monozygotic
DZ	Dizygotic
MAF	Minor allele frequency
HWE	Hardy–Weinberg equilibrium
BMI	Body mass index
AIC	Akaike’s Information Criterion
eQTL	Expression quantitative trait locus
FDR	False discovery rate
LD	Linkage disequilibrium
