# Supplementary material for: Exploratory Bivariate Genome-Wide Analysis in Northern Chinese Twins Suggests Potential Loci at 2q33.1 Harboring SPATS2L for Lung Function and Fasting Plasma Glucose
Source: Genes (Basel). 2026 Feb 24;17(3):251. doi: 10.3390/genes17030251 (PMC13025642; doi:10.3390/genes17030251)
Supplement: Supplementary file 1 [file genes-17-00251-s001.zip › Table S1.pdf]

TableS1 Basic characteristics of twin sample by sex

	variables	Male		Female		All	
		N	M (IQR)	N	M (IQR)	N	M (IQR)
Total sample	age(year)	363	50 (13.0)	391	50 (9.0)	754	50(12)
	FEV1	363	2.29 (0.90)	391	1.80 (0.67)	754	2.01(0.87)
	FVC	363	2.53 (0.92)	391	2.01 (0.65)	754	2.18 (0.90)
	FEV1/FVC	363	0.97 (0.11)	391	0.95 (0.12)	754	0.96 (0.12)
	FPG	363	5.47 (1.30)	391	4.84 (1.07)	754	5.10(1.21)
	BMI	363	24.1(4.7)	391	23.7(4.0)	754	23.9(4.3)
	Smoking status, yes	363	225(62%)	391	7(1.8%)	754	232(30.8%)
	Alcohol status, yes	363	215(59.2%)	391	22(5.6%)	754	237(31.4%)
	ALT, U/L	363	16.0(14.0)	391	20.0(14.5)	754	18(15)
	CREA, μmol/L	363	68.0(23.0)	391	75.0(27.0)	754	71(25)
	CHOL, mmol/L	363	4.78(1.66)	391	5.16(1.37)	754	4.98(1.53)
	TG, mmol/L	363	1.10(0.92)	391	1.07(0.69)	754	1.08(0.81)
	LDL, mmol/L	363	2.82(1.2)	391	2.77(1.14)	754	2.80(1.16)
	HDL, mmol/L	363	1.28(0.51)	391	1.66(0.75)	754	1.42(0.7)
GWAS sample	age(year)	141	50 (11)	137	49 (11)	278	49 (11)
	FEV1	141	2.35 (0.89)	137	1.87 (0.61)	278	2.05 (0.97)
	FVC	141	2.62 (0.93)	137	2.02 (0.55)	278	2.20 (0.90)
	FEV1/FVC	141	0.98 (0.09)	137	0.96 (0.10)	278	0.97 (0.10)
	FPG	141	5.30 (1.30)	137	4.98 (1.06)	278	5.13 (1.30)
	BMI	141	24.5(4.8)	137	23.9(4.1)	278	24.1(4.6)
	Smoking status, yes	141	85(60.3%)	137	1(0.7%)	278	86(30.9)
	Alcohol status, yes	141	86(61%)	137	7(5.1%)	278	93(33.5)
	ALT, U/L	141	17.0(16.0)	137	18.0(13.0)	278	18(15)
	CREA, μmol/L	141	70.0(23.0)	137	74.0(29.0)	278	73(27)
	CHOL, mmol/L	141	4.92(1.52)	137	5.18(1.56)	278	5.01(1.56)
	TG, mmol/L	141	1.23(1.09)	137	1.15(0.78)	278	1.2(0.92)
	LDL, mmol/L	141	2.88(1.36)	137	2.72(1.42)	278	2.77(1.42)
	HDL, mmol/L	141	1.30(0.532)	137	1.61(0.78)	278	1.42(0.69)

M (IQR)：Median（Interquartile Range）。FEV1：Forced Expiratory Volume in 1 Second; FVC：Forced Vital Capacity; FPG: Fasting Plasma Glucose; BMI: body mass index; ALT: Alanine aminotransferase; CREA: Creatinine; TC: Total cholesterol; TG: Triglycerides; LDL: Low-density lipoprotein cholesterol; HDL: High-density lipoprotein cholesterol.
