# Supplementary material for: Exploratory Bivariate Genome-Wide Analysis in Northern Chinese Twins Suggests Potential Loci at 2q33.1 Harboring SPATS2L for Lung Function and Fasting Plasma Glucose
Source: Genes (Basel). 2026 Feb 24;17(3):251. doi: 10.3390/genes17030251 (PMC13025642; doi:10.3390/genes17030251)
Supplement: Supplementary file 1 [file genes-17-00251-s001.zip › Table S5.pdf]

Table S5 Nominal significance SNPs ($P < 0.05$) of FEV1/FVC ratio-FPG in UK Biobank validation analysis

				**Discovery**		**Validation**		**Gene***	**Enhancer**
**SNP**	Chr	BP	Band	Beta	*P*-value	Beta	*P*-value		
rs296801	2	201142812	q33.1	-0.442	2.38E-07	-0.129	2.28E-02	*SPATS2L*	enh59005
rs10931896	2	201148076	q33.1	-0.457	9.37E-08	-0.127	2.43E-02		
rs295118	2	201144004	q33.1	-0.446	1.53E-07	-0.128	2.46E-02	*SPATS2L*	enh59005
rs295117	2	201146828	q33.1	-0.448	1.41E-07	-0.128	2.49E-02		
rs10497859	2	201144446	q33.1	-0.457	9.51E-08	-0.127	2.50E-02	*SPATS2L*	enh59005
rs4672726	2	201149413	q33.1	-0.457	9.51E-08	-0.108	4.56E-02		
rs11691757	2	201148951	q33.1	-0.457	9.68E-08	-0.112	4.79E-02		

*: Genes possibly regulated by Enhancers
