# Supplementary figures and images for: Exploratory Bivariate Genome-Wide Analysis in Northern Chinese Twins Suggests Potential Loci at 2q33.1 Harboring SPATS2L for Lung Function and Fasting Plasma Glucose
Source: Genes (Basel). 2026 Feb 24;17(3):251. doi: 10.3390/genes17030251 (PMC13025642; doi:10.3390/genes17030251)

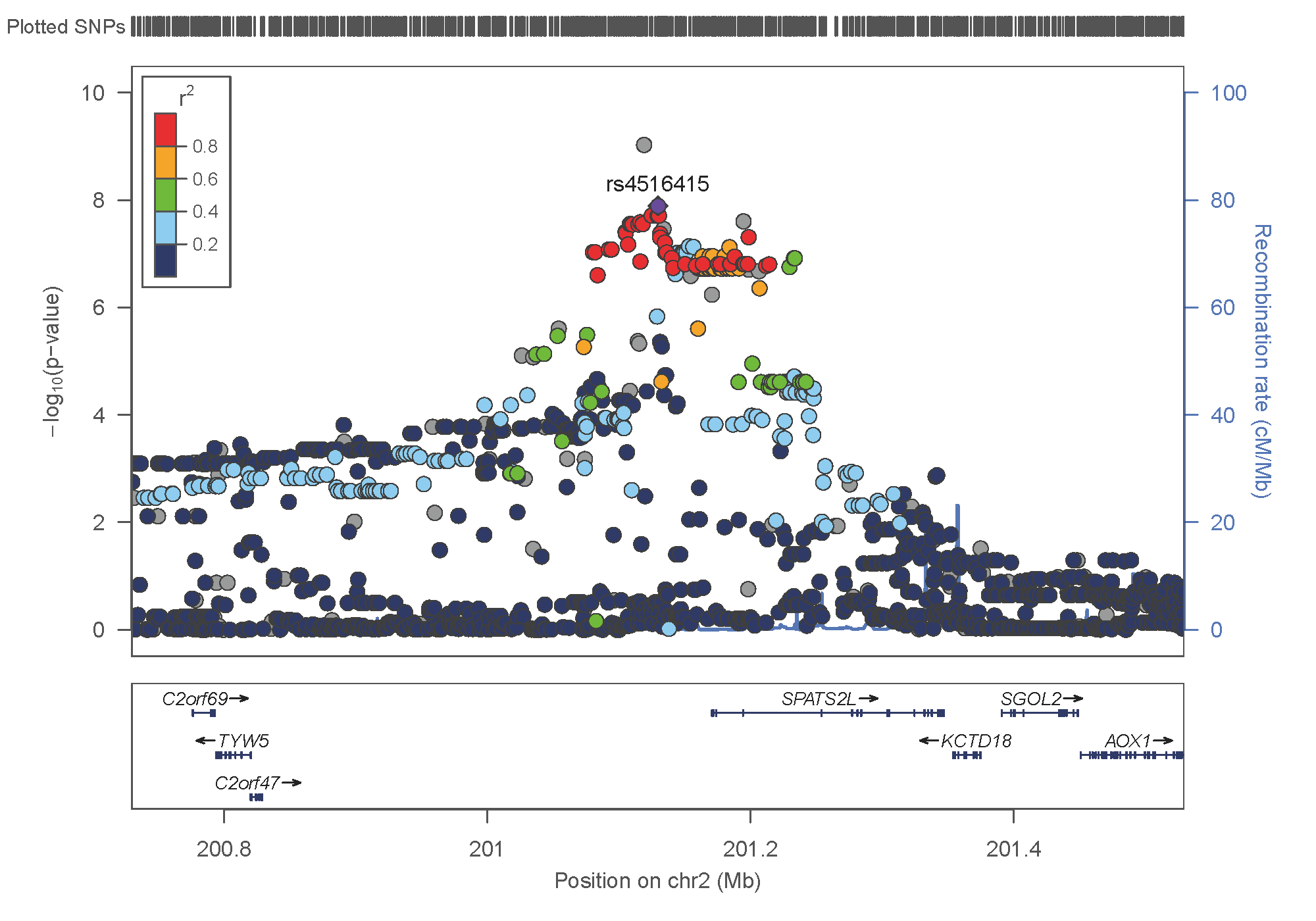

Supplement: Supplementary file 1 [file genes-17-00251-s001.zip › Figure S1.tiff]

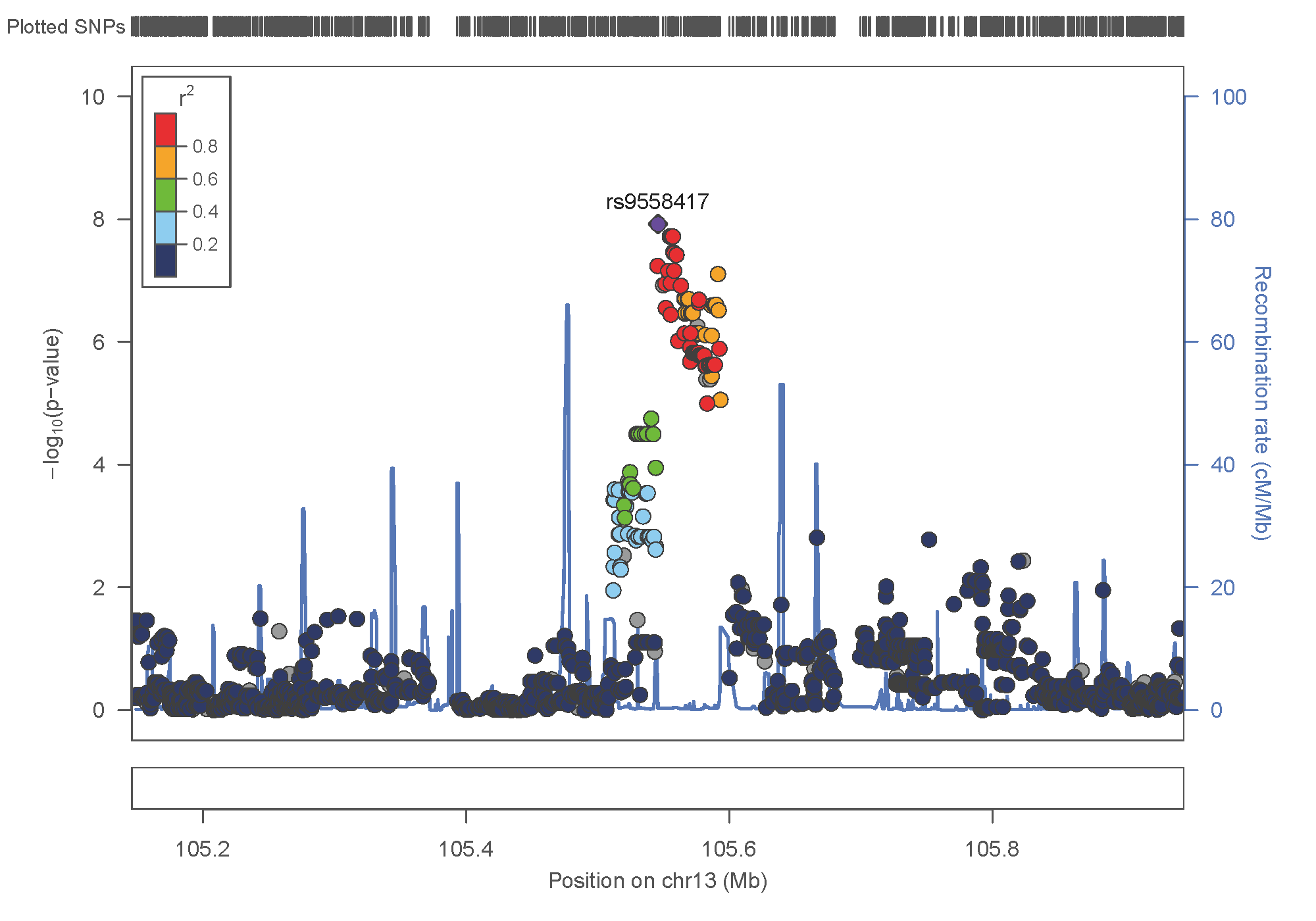

Supplement: Supplementary file 1 [file genes-17-00251-s001.zip › Figure S2.tiff]
